# Morphological and Physiological Characteristics of Laminar Cells in the Central Nucleus of the Inferior Colliculus

**DOI:** 10.3389/fncir.2012.00055

**Published:** 2012-08-20

**Authors:** Mark N. Wallace, Trevor M. Shackleton, Alan R. Palmer

**Affiliations:** ^1^MRC Institute of Hearing Research, Medical Research CouncilNottingham, UK

**Keywords:** inferior colliculus, microcircuits, fibro-dendritic laminae, flat cells, juxtacellular labeling, neuronal reconstruction, intrinsic axon

## Abstract

The central nucleus of the inferior colliculus (IC) is organized into a series of fibro-dendritic laminae, orthogonal to the tonotopic progression. Many neurons have their dendrites confined to one lamina while others have dendrites that cross over a number of laminae. Here, we have used juxtacellular labeling in urethane anesthetized guinea pigs to visualize the cells with biocytin and have analyzed their response properties, in order to try and link their structure and function. Out of a sample of 38 filled cells, 15 had dendrites confined within the fibro-dendritic laminae and in 13 we were also able to reconstruct their local axonal tree. Based on dendritic morphology they were subdivided into flat or less flat; small, medium, or large; elongated or disk-shaped cells. Two of the elongated cells had many dendritic spines while the other cells had few or none. Twelve of the cells had their local axonal tree restricted to the same lamina as their dendrites while one cell had its dendrites in a separate lamina from the axon. The axonal plexus was more extensive (width 0.7–1.4 mm) within the lamina than the dendrites (width generally 0.07–0.53 mm). The intrinsic axons were largely confined to a single lamina within the central nucleus, but at least half the cells also had output axons with two heading for the commissure and five heading into the brachium. We were able to identify similarities in the physiological response profiles of small groups of our filled cells but none appeared to represent a homogeneous morphological cell type. The only common feature of our sample was one of exclusion in that the onset response, a response commonly recorded from IC cells, was never seen in laminar cells, but was in cells with a stellate morphology. Thus cells with laminar dendrites have a wide variety of physiological responses and morphological subtypes, but over 90% have an extensive local axonal tree within their local lamina.

## Introduction

The inferior colliculus (IC) integrates projections from lower auditory nuclei with descending projections from the thalamus and cortex. Despite this pivotal position in the auditory pathway the intrinsic wiring for the IC has still not been fully described in any species. The central nucleus of the IC (CNIC) is made up of layers of neurons with their dendrites oriented in parallel, forming a series of 10–12 laminae (see Morest, 1964; Rockel and Jones, 1973; Oliver and Morest, 1984; Malmierca et al., 1995). Recent work suggests that pathways from the lower brainstem may remain functionally segregated at the IC (e.g., Davis, 2002) with terminals from different input pathways interleaved, terminating in different sublaminae within the central nucleus (Oliver et al., 1997). Descending projections from the cortex mainly terminate in the dorsal or external cortex of the IC (Coomes et al., 2005), but influence the functional properties of cells in the CNIC (Palmer et al., 2007; Wu and Yan, 2007; Nakamoto et al., 2008, 2010), presumably via intrinsic connections.

Histological studies using Golgi staining have demonstrated a limited number of separable morphological cell classes in the IC. One of the earliest studies (Oliver and Morest, 1984) defined two cell types, some of which (“disk-shaped” cells) have dendrites and probably axons that are flattened in extent so as to remain within one lamina. Other (“stellate”) cells have dendrites and axons that cut across two or more laminae and may integrate inputs from different sources. More recently, Malmierca et al. (1993) described two types of cell that contributed to the laminar organization “flat” (F) and “less flat” (LF). However, *in vitro* intracellular recordings in brain slice preparations have demonstrated at least six different cell types in the IC (Peruzzi et al., 2000; Ono et al., 2005), but the discharge patterns did not correspond simply to disk-shaped (flat) or stellate (LF) categories (Oliver et al., 1991). In a landmark study, Oliver et al. (1991) reconstructed the morphology of cells in the IC after filling with HRP. What is very clear from that study is that there is a widespread ramification of local axons within the IC and that the orientation of the soma, dendrites, and axonal fields depend upon the cell type and the position within the whole IC: within the central nucleus there were cells that based on dendrite and axon orientation seemed to correspond to the disk-shaped and stellate distinction. However, we do not know if there are also differences in the way these cell types respond acoustically *in vivo*.

In this study of the intrinsic wiring and responses of IC neurons we used juxtacellular labeling (see Palmer et al., 2003; Arnott et al., 2004) that allowed us to measure physiological response profiles of single cells, to dye fill them, and to recover their morphology. In an attempt to reduce the heterogeneity, we report the physiological characteristics of one group of cells that, superficially at least, have similar morphologies with axons and dendrites restricted to within a single frequency band lamina.

## Materials and Methods

Experiments were carried out using male and female pigmented guinea pigs ranging from 350 to 884 g. Experiments were performed in accordance with a project license issued under the United Kingdom Animals (Scientific Procedures) Act 1986. All reagents were obtained from Sigma, except where otherwise stated.

### Animal preparation

Animals were anesthetized with urethane (0.9 g kg^−1^ i.p., in 20% solution in 0.9% saline) and Hypnorm (0.2 ml i.m., comprising fentanyl citrate 0.315 mg ml^−1^ and fluanisone 10 mg ml^−1^). Atropine sulfate (0.06 mg kg^−1^ s.c.) was administered at the start of the experiment. Anesthesia was supplemented, on indication, with further doses of Hypnorm (0.2 ml i.m.). A tracheotomy was performed, followed by bilateral exposure of the ear canal by removal of the tragus and adjacent tissue. The animal was mounted in a stereotaxic frame in which the ear bars were replaced with plastic speculae to allow visualization of the tympanic membrane and delivery of sound stimuli. Pressure equalization within the middle ear was achieved by a narrow polythene tube (0.5 mm external diameter) sealed into a small hole in the bulla on each side. The angle of the head was adjusted such that the surface of the skull in the rostro-caudal axis was horizontal at points 5 and 13 mm in front of ear-bar zero (see Rapisarda and Bacchelli, 1977). Craniotomies were performed on both sides, extending 2–3 mm rostral and caudal of the interaural axis, and 3–4 mm lateral from midline. Following removal of the dura, the exposed brain was covered with 1.5% agar.

The animal’s temperature was maintained at 38°C throughout the experiment by means of a heating blanket which was thermostatically controlled using a rectal thermistor. The animals were artificially respired with 100% oxygen and the respiratory rate and end-tidal CO_2_ was monitored via an infra-red capnometer; heart rate was monitored via a pair of electrodes inserted into the skin to either side of the animal’s thorax (both Vetspecs VSM8, Canton, GA, USA).

### Stimulation, recording, and juxtacellular labeling

Experiments were carried out in a sound-attenuated booth. Stimuli were delivered through a sealed acoustic system comprising custom-modified Radio Shack 40-1377 tweeters (M. Ravicz, Eaton Peabody Laboratory, Boston) that coupled to damped 4 mm-diameter probe tubes, which fitted into the speculum. In every experiment, the sound system close to the tympanic membrane was calibrated using a Brüel and Kjaer 4134 microphone fitted with a 1-mm probe tube. The sound system response on each side was flat to within ±10 dB from 100 to 35,000 Hz.

Stimuli used in this study consisted of 50 ms bursts of tones and wideband noise (bandwidth 0.1–50 kHz), which were presented to either or both ears every 200 ms. All stimuli were generated by an array processor (Tucker-Davis Technologies AP2) housed in a computer. The stimuli were output via a waveform reconstruction filter and digital-to-analog converter at rates of at least 100 kHz. The maximum output level was set to approximately 100 dB SPL.

Recordings were made with stereotaxically placed aluminosilicate glass capillary microelectrodes (1.0 mm outer diameter with filament, Clarkes SM100F-10, Harvard Apparatus Ltd., Edenbridge, UK), pulled, filled with 1.5% biocytin in 0.5 M sodium chloride and broken back to give a tip impedance of 15–30 MΩ and advanced by a piezoelectric motor (Burleigh Inchworm, IW-711-00) into the IC through the intact cortex. Extracellular action potentials were amplified (Axoprobe 1A, Axon Instruments, Burlingame, CA, USA), filtered (300–2,000 Hz), and monitored via an oscilloscope and a loudspeaker. Action potentials were discriminated using a level-crossing detector and converted into voltage pulses which were recorded digitally with a resolution of 1 μs (Tucker-Davis Technologies SD1 and ET1).

When a single cell was isolated, using a search stimulus of diotic broadband noise or pure tones of variable level, the cell’s characteristic frequency (CF) and minimum threshold for response were determined audio-visually. The cell responses were then characterized physiologically using a battery of stimuli delivered to either or both ears as appropriate. The battery consisted of the following:

1. Frequency response areas measured in pseudo-random order by presenting single tones over a 3 octave by 100 dB range in steps of 1/8 octave and 5 dB.
2. Frequency response areas as above with a CF tone simultaneously presented at 10 dB above threshold. This revealed inhibitory areas in those neurons with low rates of spontaneous activity.
3. Rate-level functions to 10 repetitions of CF tones presented separately to the left and right ears and to both ears. The tones were presented in pseudo-random order over a 100-dB range in 5 dB steps.
4. Peristimulus time histograms of responses evoked by 150 repetitions of CF tones presented to each ear separately and to both. The tones were delivered at 20 dB above the CF response threshold.
5. Modulation transfer functions from 10 to 1,000 Hz modulation rate (11 logarithmic spaced values) measured using 200 ms of 100% amplitude modulated CF tones presented at 20 dB above threshold to both ears once every 0.8 s for 10 repetitions.
6. Interaural Level Difference (ILD) functions measured by setting the contralateral tone to 20 dB above CF threshold and varying the level of the ipsilateral tone over a range of ±20 dB above and below that level in 2 dB steps for 10 repetitions. In some experiments ILD functions were also obtained by setting the average level to 20 dB above threshold and varying both the contra and ipsilateral levels symmetrically over a ±20 dB range in 2 dB steps.
7. Interaural Time Difference (ITD) functions (only when the CF was below about 1.5 kHz) to 20 repetitions of 20 dB suprathreshold CF tones. The ITD was varied in pseudo-random order over 31 steps of 0.1 of the period of the CF tone.

Following physiological characterization, the cell was labeled with biocytin according to the juxtacellular method of Pinault (1996). Briefly, biocytin was ejected from the recording pipette under physiological control using +3 to +11 nA square wave current pulses, of 200 ms duration (50% duty cycle), which were applied using the current injection circuit of the microelectrode amplifier. Adequate current injection, appropriately close to the cell, caused action potentials to be evoked robustly during the depolarizing epochs. The current strength was titrated carefully to ensure that the cell remained firing throughout the labeling, but was not damaged by over driving; labeling occurred when current injection-associated firing was maintained for 2–15 min. We gained the impression that the duration of action potential entrainment, preferably using currents in the range +5 to +10 nA, was the most significant factor governing the distance through which a neuron might, subsequently, be traced. In three of the first cells we labeled (325L, 351L, and 385R) the spike was still well-isolated at the end of the current injection and we repeated the frequency response analysis to confirm that the cell’s physiological properties were not altered by the 10 or 15-min of driving. However, in seven of the cells we either lost the spike or it faded away during the injection and in two of the cells (505L and 939L) we recorded a burst of spikes that indicated damage associated with an injury potential. Most of the cells which showed a distinctive injury potential were never recovered. Thus, in the later experiments, we carefully backed off from the cell immediately after the end of the injection period to avoid any risk of damage.

### Histology

Following up to 9 h survival (during which time we undertook recordings from the IC using tungsten-in-glass microelectrodes (Bullock et al., 1988) for a number of different studies) the animal was anesthetized deeply with an overdose of sodium pentobarbitone. The animal was then perfused transcardially with 250 ml 0.1 M phosphate buffer pH 7.4 (PB) followed by 500 ml PB containing 4% paraformaldehyde and 0.5% glutaraldehyde. The brain was removed and stored in the same fixative overnight at 4°C.

The following day, the brain was embedded in a mixture of gelatine and egg albumin and serial 50 μm coronal or horizontal sections were cut using a vibratome. The freely floating sections were washed twice in PB and were incubated overnight at 4°C in PB containing 0.3% Triton X-100 and avidin-biotin peroxidase complex (ABC Elite, Vector Laboratories). The sections were washed twice in PB before being incubated for 10 min with 0.05% diaminobenzidine (DAB), 0.005% hydrogen peroxide, 0.0015% nickel ammonium sulfate, and 0.0015% cobalt chloride in PB. After quenching the reaction with excess PB, and washing the sections twice, the sections were mounted on subbed slides.

### Anatomical reconstructions

**Figure 1:**
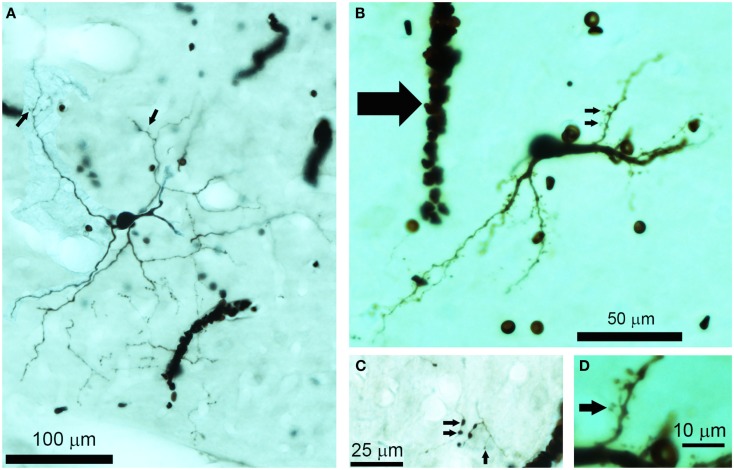
**Photomicrographs of biocytin labeled cells and processes in the IC**. **(A)** Photomontage of a large, flat disk cell (1079R) viewed in the horizontal plane. This cell only has a few sparse dendritic spines on its distal dendrites (small arrows). Red blood corpuscles are also stained and partially obscure a few of the processes. **(B)** Flat, medium elongated cell (339L) sectioned in the coronal plane. This has numerous dendritic spines even on its proximal dendrites (small arrows). Near the soma there is an electrode track that filled up with blood during the removal of the electrode (large arrow). **(C)** The disk cell shown in **(A)** had an axon with numerous axonal swellings some of which were quite large (2 μm diameter) as indicated by the pair of small arrows, but nearby there were other small axonal endings (<1  μm) as indicated by the single small arrow. **(D)** A higher power view of the dendritic spines indicated by the lower small arrow in **(B)**. These are only about 20 μm from the soma and have a long thin stalk.

**Figure 2:**
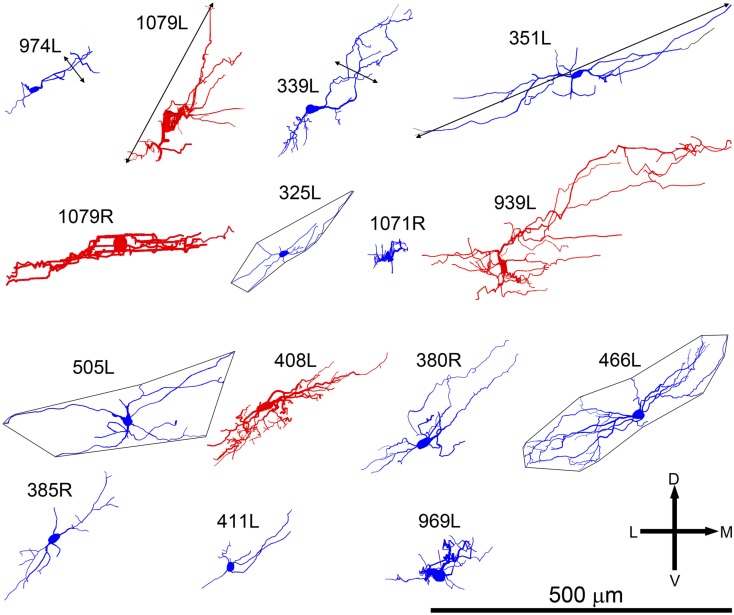
**Soma and dendrites of all 15 filled cells viewed in the coronal plane**. Most cells were filled on the left (L suffix) but some were filled in the right IC (R suffix) and these have been reversed so that all the cells appear as though they were on the left. All of the cells are flattened and their long axis is oriented approximately in the plane of the fibro-dendritic laminae. They are arranged in tonotopic order with the cell having the lowest CF (300 Hz) located in the top left corner (974L) and the cell with the highest CF (5.1 kHz) near the bottom right corner. Their “flatness” was measured by the thickness of their dendritic tree in or close to the coronal plane as illustrated by the double-headed black arrow for cells 974L and 339L. Their size was taken as the maximum dendritic extent measured in either the coronal or horizontal planes as illustrated by the double-headed arrow for cells 1079L and 351L. The area of the dendritic tree was measured in either the coronal or horizontal plane) by drawing a polygon round the main dendrites as illustrated for cells 325L, 505L, and 466L. They range in size from small, <20,000 μm^2^ to large, >45,000 μm^2^ with just under half (7/15) classed as medium.

Three-dimensional reconstructions were undertaken using computer software (Neurolucida, Microbrightfield, Colchester, VT, USA) connected to a microscope (Axioskop2, Carl Zeiss) with a motorized stage. DAB-stained material was traced using a 40× objective lens (NA 0.95) within the traced boundaries of the IC in each section. It was not usually possible to see the electrode tracks except when blood had infiltrated them as in the track shown in Figure 1B (thick arrow), which was located 50 μm lateral to the track (not visible) where cell 339L was labeled. The “flatness” of the filled cell was obtained by measuring the short axis of the dendritic tree in or close to the coronal plane as illustrated by the double-headed arrow for cells 974L and 339L in Figure 2. The maximum dendritic extent was also measured in either the coronal or horizontal planes (whichever was longer) as illustrated for cells 1079L and 351L in Figure 2. The area of the dendritic tree was measured in either the coronal or horizontal plane (whichever was larger) by drawing a polygon round the main dendrites as illustrated for cells 325L, 505L, and 466L in Figure 2. The borders of the different divisions of the IC are difficult to discern in the guinea pig even when sections have been counterstained for Nissl substance and the borders mainly seem to be gradual transitions rather than having a sharp edge. We therefore estimated cell positions based on comparisons with a standard series of sections stained for nitric oxide synthase (Coote and Rees, 2008). The strength of staining in the dendrites varied between cells, but as we could distinguish dendritic spines in 10/15 cells, even when the staining was relatively weak, we were confident that we were able to follow most of the dendrites to their end.

## Results

### Dendritic morphology of laminar cells

In our study of the IC we filled and recovered 38 individual cells. Some of these were in the external or dorsal cortices and of those in the central nucleus some had dendritic trees that were oriented across the laminae or were big enough to extend over two or more laminae (stellate cells). This left 15 cells which had dendrites oriented along the plane of a lamina in the central nucleus and where the dendrites appeared to be restricted to one lamina. Some of the cells were less flat than others and the thickness (dorso-ventral) of the dendritic trees within the lamina varied from 20 to 135 μm. Thus nine of the cells had a flat morphology (dendritic thickness =80 μm viewed in the coronal plane) while six were LF. All were considered to be laminar cells because all their major dendrites were primarily oriented in the plane of their intrinsic axon which we assumed formed part of a single lamina. In practice, all the cells had dendritic trees where the longest dimension within the lamina was at least twice that of the thickness across the lamina and the mean ratio of these two dimensions was 4.7 (range 2–7.4) as shown in column 11 of Table 1. These 15 cells form the basis of the current report and examples of the soma and dendrites of two of them are shown in Figure 1. Although we defined the laminar cells as a single class, the characteristics of their dendrites meant that the cells could be subdivided on the basis of flatness, dendritic area, orientation, symmetry, and number of spines (Table 1).

**Table 1:** **Morphological characteristics of the 15 filled cells**.

Cell no.	CF (kHz)	Soma Area (μm^2^)	Dendrites Area (μm^2^)	Dendritic morphology						Dendrites (μm) min/max diameter	Local axon	Projection
				Shape of tree			Spines	Orientation	
974L	0.297	133	9,777	Flat	Small	Elongated	Sparse	A	M/L	80 × 162	Incomplete	2
1079L	0.669	308	32,383		Medium	Disk	Sparse	A	M/L	125 × 272	Incomplete	2
339L	0.933	185	24,994	Flat	Medium	Elongated	Many	A	Oblique	50 × 364	Laminar	Commissural
351L	0.947	205	48,412		Large	Elongated	Sparse	S	M/L	90 × 574	Laminar	2
1079R	0.983	243	90,371	Flat	Large	Disk	Sparse	S		70 × 395	Laminar	Brachium
325L	0.991	130	7,260	Flat	Small	Elongated	1	S	M/L	20 × 141	Unstained	2
1071R	1.144	62	7,583	Flat	Small	Elongated	Many	S	R/C	75 × 253	Laminar	Intrinsic 2
939L	1.377	214	133,235		Large	Disk	1	A	M/L	135 × 612	Laminar	Brachium
505L	1.469	264	27,568		Medium	Elongated	1	S	M/L	100 × 373	Laminar	2
408L	2.193	193	32,887	Flat	Medium	Disk	Sparse	S		60 × 306	Laminar	Commissural
380R	2.332	213	24,016	Flat	Medium	Elongated	Sparse	A	M/L	45 × 331	Laminar	Brachium 2
466L	3.303	218	34,754		Medium	Elongated	Sparse	S	M/L	100 × 396	Laminar	Brachium
385R	4.497	248	14,350	Flat	Small	Elongated	Sparse	S	M/L	60 × 281	Unstained	2
411L	4.929	118	7,230	Flat	Small	Elongated	1	A	Oblique	60 × 195	Laminar	Intrinsic
969L	5.119	402	37,337		Medium	Elongated	1	S	R/C	100 × 343	Laminar	Brachium

*A, asymmetric; S, symmetric; M/L, medio-lateral; R/C, rostocaudal. ^1^The staining was too weak to be certain whether spines were present or not. ^2^Uncertain on where the cells project to*.

All the cells had a similar orientation when viewed in the coronal plane (Figure 2). In this figure the cells are illustrated as though they were all from the left IC and all have an orientation running from ventro-lateral to dorso-medial. The exact orientation varies, with the long axis varying between about 10° and 45° to the horizontal. This range of orientations is also found in the fibro-dendritic laminae visualized by the course of stained groups of intrinsic or commissural axons (Malmierca et al., 1995) and there seems no doubt that these 15 filled cells form part of the laminae. The cells were all multipolar with three to seven primary dendrites. Two of the cells had quite dense dendritic spines even on the proximal dendrites whereas the rest had either no spines or they were sparsely located on the distal dendrites (Figure 1). Most cells had well-stained dendrites and we were confident that any spines present would have been stained. However, five cells had weakly stained dendrites and the absence of spines may have been due to inadequate staining (see Table 1).

Most of the axonal swellings observed were “en passant” although there were some terminal boutons as well. The size of these swellings varied from 1 to 2 μm and varied along the length of an axonal branch (Figure 1C) so that small and larger boutons were present close to each other. We made no attempt to plot the distribution of axonal swellings or determine if a particular subtype of laminar cell was associated with a higher proportion of large axonal swellings and so this remains a possibility that may be worth pursuing in future.

**Figure 3:**
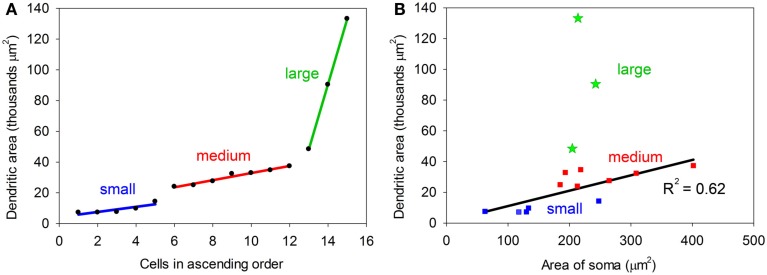
**(A)** Plot showing the dendritic areas of the 15 labeled cells arranged in ascending order. Regression lines have been plotted for each of the three groups which have been defined by a break in the slope of the plot. **(B)** Plot relating the area of the cell body to the area of the dendritic tree. For the small and medium cells there is a reasonably good correlation (*R*^2^ = 0.62), but for the large cells (green) the two values are not correlated.

One obvious difference is in the extent of the dendritic spread with the maximum area of the dendrites in some cells being less than 20,000 μm^2^ (small) and over 45,000 μm^2^ in others (large). The medium cells have an intermediate range of values and when dendritic area is plotted in ascending sequence the values seem to fall into three distinct groups (Figure 3A). However the small sample size means that these groups are a bit arbitrary and we cannot conclude that there are three discrete groups in any absolute sense. The cross-sectional area of the somata also varied with values ranging from 62 to 402 (μm^2^). The area of the soma and the area of the dendritic field were roughly proportional for the small and medium cells as shown in Figure 3B. When a regression line is plotted for these two groups the correlation value is reasonably high, *R*^2^ = 0.62. By contrast, there is a definite lack of correlation between the area of the soma and the dendritic area for the three large cells as their somata are the same size as the smaller medium cells. This indicates that in histological preparations where only the somata are stained it cannot be assumed that a larger soma automatically means a larger dendritic tree. In other neuronal systems the cell body size is proportional to the length of the axon (Ho et al., 1992) and this may also be true in the IC.

**Figure 4:**
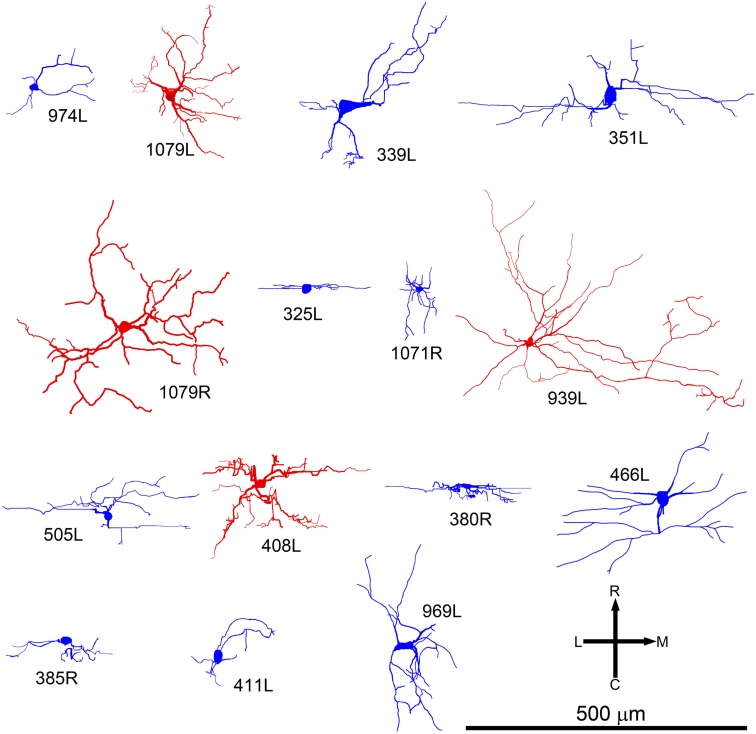
**Soma and dendrites of all the cells shown in the same tonotopic order as in Figure 2 but viewed in the horizontal plane**. At this orientation many of the cells still have an elongated cylindrical shape (blue cells). Others have a more stellate appearance and are classed as disk cells (red).

When the cells were viewed in the horizontal plane it was possible to visualize the extent of the dendrites within what we assume was the plane of a single lamina (Figure 4). In this orientation there were striking differences in the shape of the dendritic tree with four of the cells having dendrites radiating out in at least four directions (disk cells) while the other 11 cells had their dendrites arranged primarily along a single axis to give an elongated shape. Seven of the elongated cells were oriented in the medio-lateral direction, two in the rostro-caudal direction, and two were obliquely oriented (Table 1). Some of the disk cells and elongated cells were reasonably symmetric, but others had clear asymmetries with a greater number or length of dendrites oriented in one direction than in the opposite direction.

### Spatial extent of intrinsic axons

**Figure 5:**
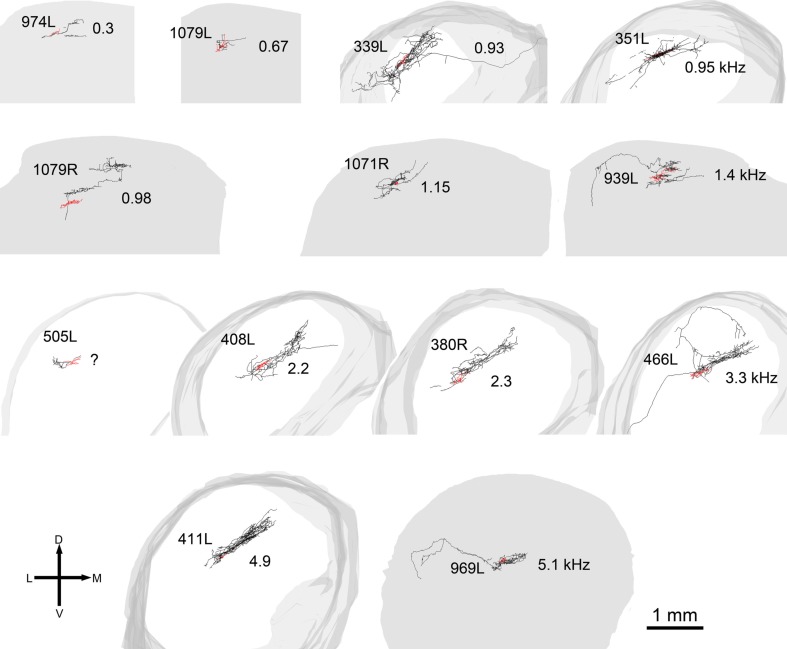
**Reconstructions of the axons and dendrites of filled cells viewed in the coronal plane**. The axons are black, the dendrites are red, and the shape of the IC is represented in gray. In some cells the axon has been incompletely filled and sections are missing (e.g., 974L) while for two of the cells the axon was not stained and these cells are not shown. The cells are arranged in the same tonotopic order as in Figure 2 and the CF of the cell (in kHz) is shown to the right of each cell except for 505L where we had two physiological response profiles recorded and could not determine which one corresponded to the filled cell.

**Figure 6:**
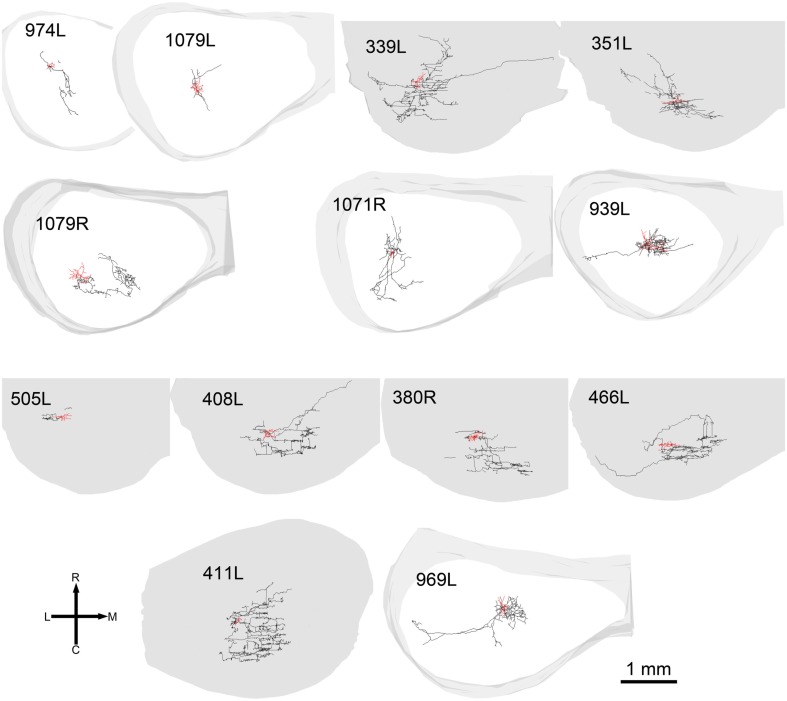
**Reconstructions of axons and dendrites of filled cells in the same format and order as in Figure 4 but viewed in the horizontal plane**.

**Figure 7:**
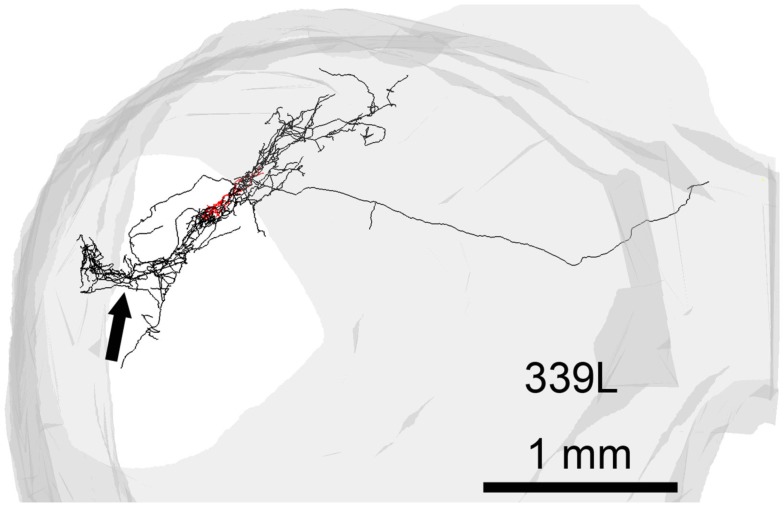
**Reconstruction of the axon (black) and dendrites (red) of a single cell (339L) viewed in an oblique plane optimized to show how the branches of the intralaminar intrinsic axon bend round to follow the upward sweep of the lamina at the lateral edge (black arrow)**.

In 13 of the cells the labeling was sufficiently good that we were able to reconstruct much of the intrinsic axon (see Figure 5, axons in black, dendrites in red). When viewed in the coronal plane, 12 of these cells had intrinsic axonal trees that were more extensive than the dendrites, but confined to the same lamina. Some also appeared to have an extrinsic axon with two cells sending an axon toward the commissure and five separate cells sending an axon toward the brachium. Two cells had relatively small axonal trees that were also mainly in the same lamina as the dendrites, but were incompletely stained. However, one cell (1079R) had an intrinsic axon that appeared to be confined to a separate lamina from that in which the dendrites lay. When viewed in the horizontal plane it was possible to see the whole extent of each axon within a lamina (Figure 6). There was no apparent correlation between the extent of the intrinsic axonal tree and that of the dendrites. One of the small, flat elongated cells (411L) had one of the biggest axonal extents, while the other small, flat elongated cell (1071R) had a less extensive tree. Similarly, the large disk cell (939L) had one of the smallest axonal trees, while the other large disk cell (1079R) had a more extensive axonal tree. The pattern of the intrinsic axon seemed quite specific both in terms of extent and orientation of the axon terminals. Some cells (e.g., 408L) had axons oriented in the medio-lateral direction, some (e.g., 1071R) in a rostro-caudal direction, and some (e.g., 1079R) in an oblique direction. The axons were always more extensive than the dendrites with the medio-lateral width of the dendrites being 0.07–0.53 mm and the width of the axonal tree being 0.7–1.4 mm. The axonal trees never extended across the whole width of the central nucleus, but they sometimes extended over half the width and this was true in the rostro-caudal direction as well. Cells that were placed in the lateral part of the central nucleus generally had axonal trees that were mainly located medial to the soma. In the case of one laterally placed neuron, that had axonal branches running both medially and laterally (339L), the lateral branches followed the lamina round as it turned in the dorsal direction. This is shown in Figure 7, where the reconstruction has been oriented to show how the axon terminals line up in the dorsal extension of the lamina (thick arrow).

**Figure 8:**
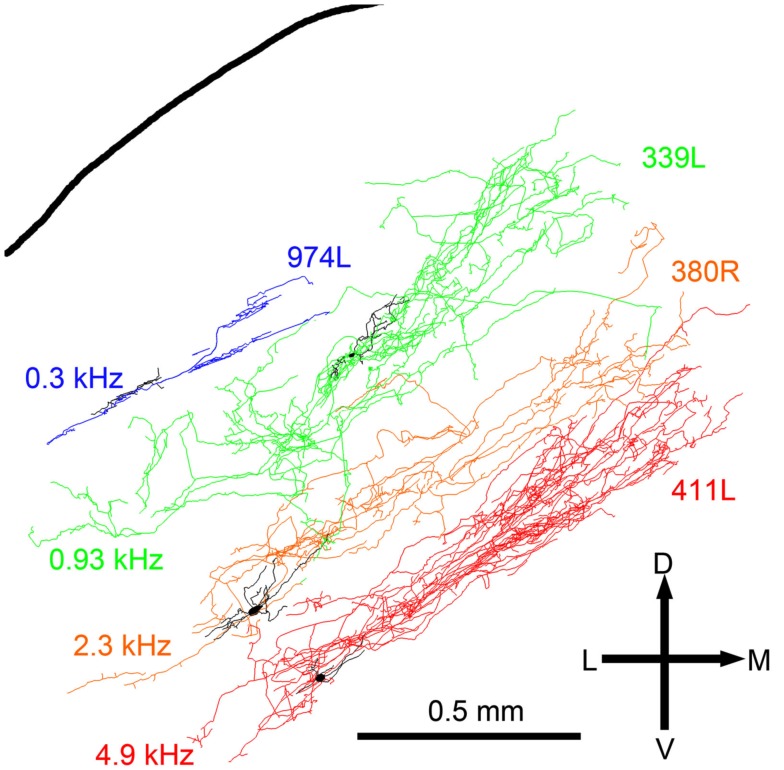
**Reconstructions of the intrinsic axons (blue, green, orange, and red respectively) and dendrites (black) of four filled cells superimposed at the appropriate depth below the IC surface and arranged in order of CF (shown to the left)**.

The laminar cells included in this report have CFs below 5.2 kHz. Their position within the IC and the orientation of their dendritic and axonal trees follow the tonotopic laminae with low frequencies more dorsally located (Figure 8). The four illustrated cells have CFs that just over 1 octave apart and there is almost no overlap in their axonal trees. For the cells with CFs of above 1 kHz the laminar thickness of their axonal tree is about 200 μm. For the low-frequency cells (CFs <1 kHz) the intrinsic axons were less extensive and there may not be such clear laminae.

### Physiological responses of laminar cells

**Figure 9:**
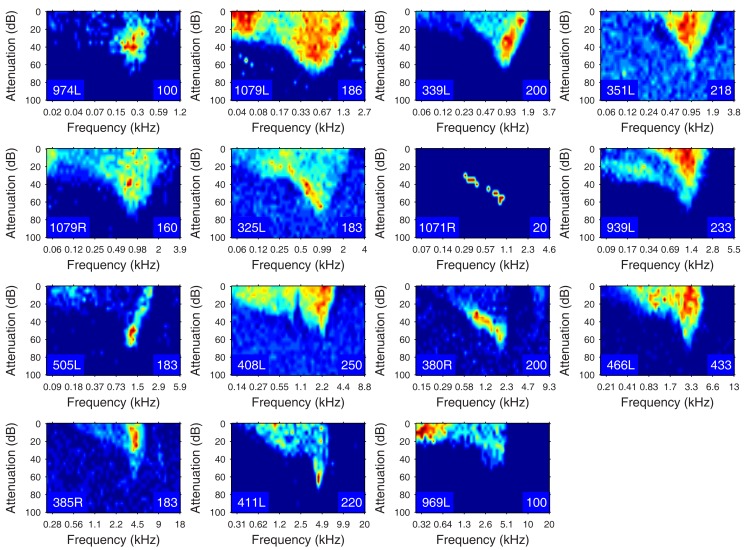
**Frequency response areas (see “Materials and Methods”) for the 15 filled cells**. The topographic arrangement follows the previous figures and is tonotopic. The response areas are normalized on a temperature scale from blue (zero response) to red (maximum response). The number in the bottom right hand corner of each panel for this and the next figure indicates the maximum firing rate to which the plot is scaled (spikes/s).

**Figure 10:**
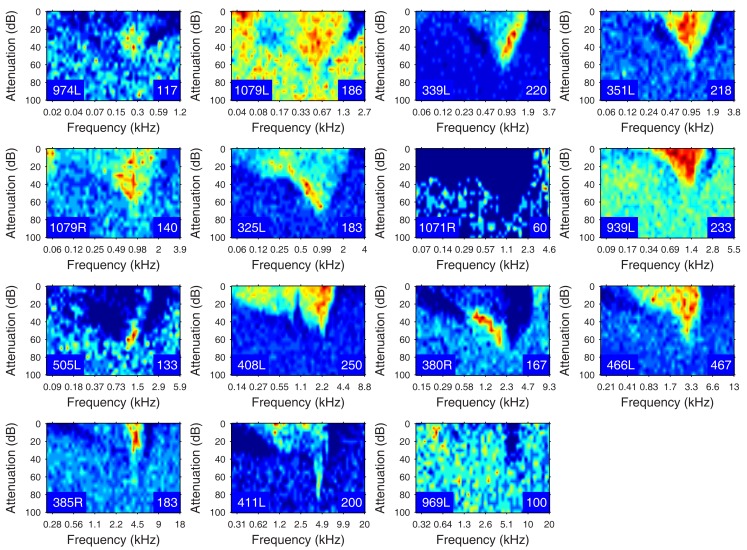
**Frequency response areas in the same format as Figure 8, but measured in the presence of a simultaneously presented CF tone at 10 dB above minimum threshold**. Units 351L, 325L, and 408L are repeated from Figure 9 since they had a sufficiently high spontaneous rate to reveal inhibition without the presence of a second tone.

**Figure 11:**
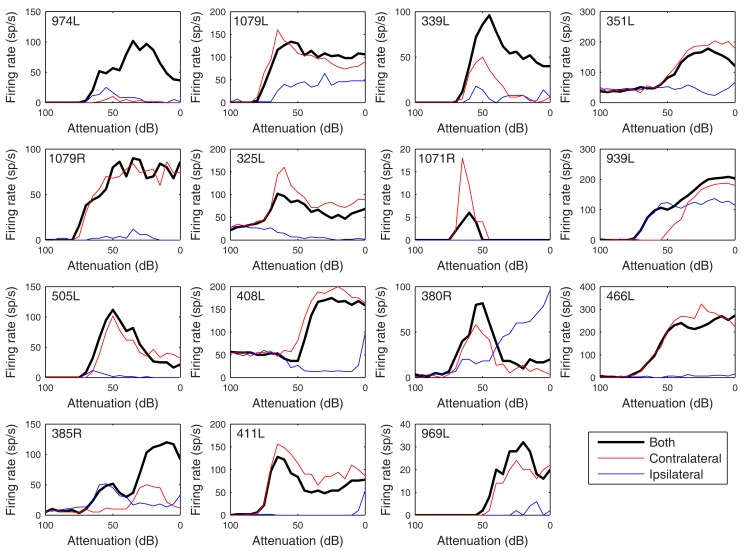
**Rate versus level functions in response to 50 ms CF tones arranged in the same order as previous figures**. The black curves show responses to binaural presentation, red to contralateral, and blue to ipsilateral.

**Figure 12:**
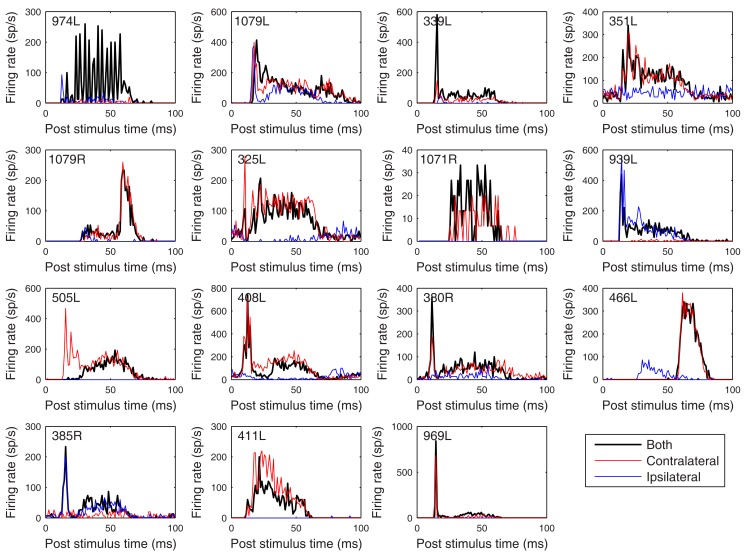
**Peristimulus time histograms to 50 ms CF tones**. The figure is arranged in the same order as previous figures with the tones presented binaurally (black), contralaterally (red), and ipsilaterally (blue) at 20 dB above minimum threshold. Binwidth is 1 ms.

**Figure 13:**
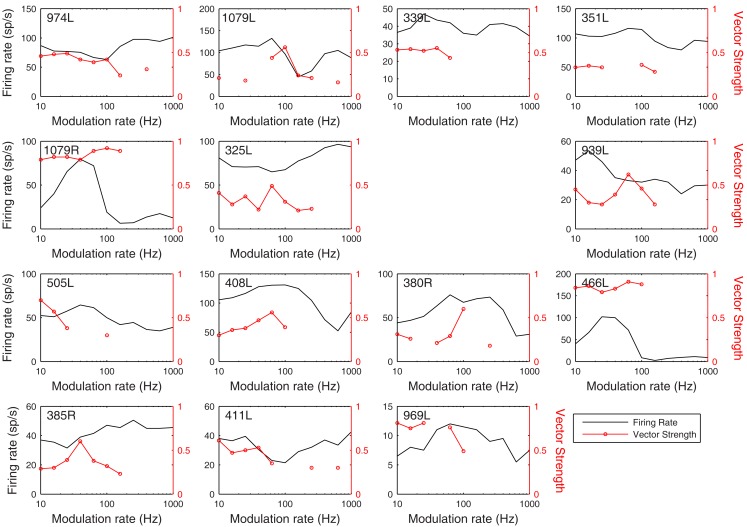
**Modulation transfer functions in response to 200 ms, 100% amplitude modulated CF tones arranged in the same order as previous figures**. The black lines show the average discharge rate in response to the modulated stimuli. The red curves show the vector strength measured from period histograms locked to the modulation waveform.

The heterogeneity of the somatic, dendritic, and axonal morphologies was also evident in the physiological response profiles recorded from each of the cells prior to dye filling. This is illustrated for five elements of the profile in Figures 9–13 and summarized in Table 2. The binaural response properties (interaural time and level difference sensitivities) did not appear to provide additional explanatory leverage in this sample and are not shown here. Each of the physiological figures is arranged in exactly the same tonotopic order as the summary morphological (Figures 2 and 4) so responses can be associated with individual cells. Figures 9 and 10 show respectively the response areas and the inhibitory areas that are revealed by simultaneously presenting a tone at the CF. It is clear that within this relatively small sample we see examples of many of the well-described response area types (“V”: 351L; “tilted”: 339L, 1071R, 505L, 380R; “O”: 974L). It is also clear that many of the cells receive extensive inhibitory input (1071R, 505L, 380R, 411L). When inhibition is evident in the single tone response area at the CF this results in non-monotonic rate-level functions as shown in Figure 11 (1071R, 505L, 380R, 411L). It is also clear from the rate-level functions that most cells receive binaural inputs, although some are monaurally dominated (Table 2). They are generally dominated by the contralateral inputs (red lines), in some instances the ipsilateral response is suppressive, reducing the binaural response below that to the contralateral alone (411L) and in others it is facilitatory (974L, 339L, 385R, 969L). The PSTHs to suprathreshold CF tones (Figure 12; Table 2) are diverse, but have one thing in common: none of them is a pure onset response. Onset responses were relatively common in our sample, with 16% (6/38) of our filled cells showing pure onset responses, but none of them had a laminar morphology and they will be described elsewhere.

**Table 2:** **Physiological characteristics of the 15 filled cells**.

Cell no.	CF kHz	RSP	Inhibitory sidebands		Histograms			Binaural response	ITD	ILD	Lat. ms
			Lower	Upper	Contra	Ipsi	Both	
974L	0.297	C	Strong	Strong	Low-sustained	Onset	Phase-locked	ee/F	Strong	Weak	12
1079L	0.669	V	Strong	Strong	Pauser	Pauser	Sustained	EE/s	Weak	Weak	16
339L	0.933	TU	Strong	Strong	Pauser	None	On-sustained	EO/F	Weak	Peak	15
351L	0.947	V	Weak	Weak	Spont.	Chop T	Chop T	EO/m	None	Moderate	15
1079R	0.983	V	Weak	Weak	Sustained+large offset	None	Sustained+large offset	eE/m	Weak	Weak	30
325L	0.991	V	None	None	Chop T	Inhibited	Chop T	EI/i		Strong	10
1071R	1.144	TD/C			Sustained	None	Sustained	EO/m	Strong		25
939L	1.377	V	Weak	Strong	Low-sustained	On-sustained	On-sustained	eE/i	None	Weak	13
505L	1.469	TU	Strong close	Strong	Pauser	None		EO/i		Strong	15
408L	2.193	V	Weak	Weak	On-sustained	Inhibited	Pauser	Ei/i		Weak	8
380R	2.332	TD	Strong	Strong	Pauser	Low-sustained	Pauser	eE/f		Weak	10
466L	3.303	V	Weak far	Strong	Offset	Sustained	Offset	Eo/m		None	58
385R	4.497	N	Strong close	Strong	Spont.	Pauser	Pauser	OE/m		Strong	14
411L	4.929	V	Strong far	Strong	Build-up	None	Build-up	EO/i		Strong	12
969L	5.119	V	Weak	Strong	Pauser	None	Pauser	EO/m		Weak	13

*C, closed; V, v-shaped; TD, tilt down; TU, tilt up; N, narrow. Histograms were defined following Le Beau et al. (1996): sustained units fired throughout the stimulus; On-sustained had a clear onset response, but also fired throughout the stimulus; Low-sustained fired at low level throughout the stimulus (relative to other conditions for that cell); Pausers fired strongly at stimulus onset then paused before firing for the rest of the stimulus; Build-ups were like pausers but without the onset component; Chop T fired regularly at the beginning of the response, but became less regular throughout the stimulus; Inhibited showed a decline in firing below spontaneous; Spont fired at the same spontaneous rate throughout the stimulus; None did not fire before or during the stimulus. Binaural responses: three letters show response: contralateral, ipsilateral/binaural: e, weak excitation; E, strong excitation; O, no response; f, weak facilitation; F, strong facilitation; s, summation; i, weak inhibition; I, strong inhibition; m, monaural response only, no binaural interaction (Irvine, 1986)*.

The cell with the lowest CF (974L) shows excellent phase locking to the CF tone, but only when presented to both ears (consistent with the rate-level response). Some of the cells show responses that exceed the duration of the CF tones (50 ms) and two showed a very prominent offset response (1079R and 466L). Latency varied between 8 (408L) and 25 ms (1071R) for all bar the two cells with strong offset responses (see Table 2).

The rate and temporal modulation transfer functions (Figure 13) were generally not very informative with two exceptions (1079R and 466L), which were the only cells that showed a relatively narrow bandpass rate response to the modulated stimuli. Note that in this sample of 14 cells none showed a pronounced tuning in their temporal responses (red lines show the vector strengths of period histograms locked to the modulation waveform) even though locking to the modulation was often very good (1079R for example).

Given the diversity of both morphology and physiology it is perhaps unsurprising that correlations between the two were not strong. Two cells from different animals had almost interchangeable response profiles: 1079R and 466L: V-shaped response areas with weak inhibitory input, monotonic contralaterally dominated, monotonic rate-level responses, very prominent offset responses and narrow bandpass average rate modulation transfer functions. Both cells were relatively large multipolar cells, but at least by our subjective criteria 1079R was disk-shaped, while the dendrites of 466L were oriented (see Figure 4) and hence we describe it as elongated. Cell 466R was a LF, sparsely spinous, medium elongated cell of which we have two other examples (505L and 969L, see Table 1). These latter cells show some similarities in response profile with each other, but not with 466R. The two flat sparsely spinous, medium elongated cells (380R and 385R, see Table 1) also share similar physiological response profiles: they have single and two-tone response areas and rate-level functions showing evidence of inhibition, pauser PSTHs, and untuned or broadly tuned responses to modulated stimuli. Two cells that appeared to show chopping at the onset to CF tones (351L, 325L) both had V-shaped response areas with sufficient spontaneous activity that we did not need to measure responses to two tones, had monotonic, contralaterally dominated rate-level functions, and untuned modulation transfer functions. However, one was a large elongated cell (351L) and the other was a flat, small elongated cell (325L). Similarly, for the two cells with on-sustained responses which also shared other response similarities, one was a flat, medium fusiform cell with many spines (339L) while the other was a large disk cell with only a few sparse spines (939L). Finally, the very smallest cells such as 325L, 1071R, 380R, 411L appeared to be subject to stronger and more widespread inhibitory inputs (see Figure 10).

It is clear that there are similarities in the response profiles between small numbers of cells in our sample and some of these seem to relate to small subgroups of the filled cells. However, given the diversity of morphological and physiological subtypes a much larger sample would be needed to be sure that these were real correlations.

## Discussion

### Definition of cell types based on dendritic morphology

The CNIC has been the subject of many morphological studies, but since a common nomenclature has not emerged that can be applied across all mammalian species, we have used descriptive terms taken from a number of studies. We followed the classification used in the cat IC, where cells in the central nucleus were divided into laminar disk cells and translaminar stellate cells (Oliver and Morest, 1984; Oliver et al., 1991). For the present purposes, we have chosen to describe only those cells in our sample that had their dendrites oriented along the axis of a single fibro-dendritic lamina. We excluded the cells which had dendrites primarily oriented across the lamina or appeared to extend beyond a single lamina. Based on a preliminary analysis we have about 10 such translaminar stellate cells in our material, but have not yet reconstructed them and are unable to describe them here. We also labeled about 12 stellate neurons in the dorsal and external cortex and one in the rostral nucleus, but will not be sure of their exact location until after we have reconstructed them.

The width of disk cells in the optimal plane, using the original definition (Oliver and Morest, 1984; Oliver et al., 1991), was no more than 70 μm, but some of our laminar cells clearly had a dendritic width that was almost double this. In their study of the rat IC Malmierca et al. (1993) defined flat cells based on complex criteria and stated that the mean width of their dendrites was 50 μm, while the mean width of the LF cells was 100 μm. However, the term disk cell is a useful descriptor and for this study we have extended the definition to include both the original “flat” and also “LF” laminar cells. We considered all the laminar cells in this study to be disk cells and divided them into flat and LF varieties. Two types of “disk-shaped” cells were also described in the squirrel monkey by Fitzpatrick (1975), but she also described a type of stellate cell which appeared to be confined to a single lamina. When viewed in the horizontal plane some (4/15) of our disk cells had a clear stellate morphology with multiple dendrites radiating out from the soma. However, the majority were clearly polarized so that most of their dendrites were oriented in one direction. It might have been appropriate to call these fusiform or bitufted cells as has been done previously in the cat central nucleus (Rockel and Jones, 1973), but we chose instead the more neutral term of elongated.

Another feature that has been used in classifying cells in the IC has been the distribution and density of their spines (Gonzalez Hernandez et al., 1986). In the auditory cortex spiny stellate cells are exclusively excitatory, while smooth or sparsely spinous stellates are inhibitory and GABAergic (Wallace and He, 2011). However, it is not clear if there is a similar correlation in the IC. It would be very useful to be able to identify GABAergic neurons based on their morphology, but that does not seem to be possible currently and inhibitory cells have to be identified by the use of immunohistochemical identification of specific markers (Ito et al., 2009) or genetic alteration to link production of a fluorescent protein to that of the synthetic enzyme for GABA (Ono et al., 2005). A high proportion of cells in the IC are GABAergic (about 20% in the cat; Oliver et al., 1994) and a similar proportion may be present in the guinea pig (Thompson et al., 1985). There are at least two morphological types of GABAergic cells in the central nucleus with the larger ones projecting to the thalamus (Peruzzi et al., 1997; Ito et al., 2009), while the smaller ones may be intrinsic. Many of these GABAergic cells are stellate (Oliver et al., 1994), but up to half of them may be laminar cells. In one study of GABAergic cells in the mouse IC (Ono et al., 2005) 17 GABAergic cells in the central nucleus were filled with biocytin to demonstrate their dendrites. Four of these appeared to be laminar cells and some of the laminar cells in our sample may also have been GABAergic. The most likely candidate was cell 1079R which was large, sparsely spinous, had an output axon that entered the brachium and was the only laminar cell to have an intrinsic axon that appeared to terminate in a different lamina. GABAergic laminar cells could provide a local inhibition of nearby cells with the same CF, of the sort that have been demonstrated in the guinea pig (Le Beau et al., 2001), since the axons are generally in the same lamina as the dendrites. It is not known if the dendrites of all GABAergic neurons in the central nucleus are smooth/sparsely spinous, but there does not seem to be any evidence that they are spiny. In a double labeling study of the connections of GABAergic neurons in the rat IC many cells with commissural axons were labeled, but none of them appeared to have many spines (Zhang et al., 1998), even though some spiny neurons are known to have commissural axons (Gonzalez Hernandez et al., 1986). Two of our filled cells had numerous spines, some of which were present on the proximal dendrites and one of these had a commissural axon. Based on Zhang et al.’s (1998) data and its aspiny nature this would indicate that it was probably excitatory. Others clearly had a lower density of spines that were more restricted to the distal dendrites. Some cells had no discernable spines, but in some of these the dendrites were only relatively weakly stained and thus any spines would probably not have been stained.

### Significance of the intrinsic axons

One of the most striking features of the cells described here was that the intrinsic axons were restricted to a single lamina: in all but one case this was the same lamina as that containing the dendrites. The presence of dense bands of intrinsic axons that form a single lamina has already been shown by small extracellular injections of tracers in the rat (Saldana and Merchan, 1992) and guinea pig (Malmierca et al., 1995), but it was not clear what cell types produced them. A previous intracellular study of cells in the cat (Oliver et al., 1991) had shown some examples of cells with laminar dendrites and axons confined to the same lamina, but the present study is the first to show how extensively individual axons can ramify within a lamina. These intrinsic laminae have similar dimensions to the laminae formed by inputs from other brainstem nuclei such as the cochlear nucleus or nuclei of the lateral lemniscus (Malmierca et al., 2005; Loftus et al., 2010) and are 150–200 μm.

The lack of an identifiable projecting axon in many of the cells in our sample is interesting. Given that we were able to stain the intralaminar axonal plexus of many of these cells extending widely from the cell body into very small axon branches and further that we were able to identify projecting axons in some of our sample, it seems unlikely that we would not have stained a major output axon. Clearly, given the obvious complexity of the axonal trees we might simply have not recognized the projecting axon, or the stain might have faded before the axon exited the lamina. However, one intriguing possibility, in line with other anatomical considerations, is that many of the response types that have been identified could be from neurons intrinsic to the IC and not necessarily represent the information that is passing from the IC to the thalamus.

The high density of intrinsic axons (and presumed synaptic contacts) shown in the present study is reminiscent of the neocortex where even in the input layer IV the density of intrinsic synapses is thought to outnumber the thalamic synapses by almost 10 to 1 (Peters and Payne, 1993). Although we have only studied laminar cells, the other types of cells in the central nucleus have also been shown to have extensive intrinsic axons (Oliver et al., 1991). As the IC is the largest subcortical auditory nucleus in the rat (Kulesza et al., 2002), and has almost five times as many auditory neurons as in the entire medulla and pons, the synapses derived from intrinsic axons are thought to greatly outnumber those from all other sources to the central nucleus (Saldana and Merchan, 2005). Given the large number of intrinsic synapses it is perhaps surprising how similar the responses of neurons in the central nucleus are to those of their brainstem inputs (McAlpine et al., 1998; Davis et al., 1999; Ramachandran et al., 1999). The extrinsic inputs create at least three functional zones in the central nucleus so that the low-frequency cells (<500 Hz) with a phase-locked response pattern and strong ITD sensitivity, such as cell 974L, almost certainly have their main extrinsic input from the ipsilateral medial superior olive (Loftus et al., 2010). When we measured the latency for the beginning of driven activity in our labeled cells it ranged from 8 to 30 ms (Table 2). This is much greater that the range of latencies typically seen among lower brainstem nuclei such as the cochlear nucleus (e.g., Palmer et al., 2003; Arnott et al., 2004) and leaves open the possibility that some IC neurons are partially or even mainly driven by intrinsic connections rather than by a direct extrinsic input alone. The significance of this is not clear, but it may make the individual cellular responses more consistent and reproducible because the form of the sustained response reflects an average of the local population rather than a more variable representation of its extrinsic inputs. The IC also receives a substantial input from the auditory thalamus and cortex, but these are thought to mainly terminate in the external nucleus and dorsal cortex (Winer, 2005).

### Correlation of morphology and physiological responses

A primary goal of the present study was to look for correlations between cellular morphology and physiological response properties, as has been successfully accomplished previously in the cochlear nucleus (Rhode et al., 1983a,b; Palmer et al., 2003; Arnott et al., 2004). Previous anatomical and physiological studies of the IC have revealed considerable diversity, but have generally summarized their findings in terms of a small number of identifiable classes whether of response type or morphology. Thus, in some studies only a few different frequency response areas have been recognized (e.g., Ramachandran et al., 1999; Davis, 2005), only two major morphological types have been recognized in another (Morest and Oliver, 1984), and only six response types have been described in brain slices (Peruzzi et al., 2000). Given these relatively small numbers it seemed entirely reasonable that one should be able to associate the physiological response profiles with the underlying morphological response types. Certainly, our understanding of the cochlear nucleus wiring pattern was enormously advanced by the pioneering studies correlating structure and function (Rhode et al., 1983a,b). However, early in our analysis it became clear that we, like many others, were seeing considerable diversity in both the physiological response profiles (see for example Irvine, 1986; Le Beau et al., 1996) and in the morphology: despite the fact that nearly all cells had a multipolar shape they clearly did not appear to fall into a small number of homogeneous groups.

Nevertheless, there are some possible correlations that might be drawn between the present data and the six classes of response shown in the slice preparation. A vast majority of the responses in the slice were sustained with pause or build-up temporal response patterns and such patterns are readily seen in the PSTHs in Figure 12 (505L, 408L, 380R, 385R, 969L), where all of our sample had sustained responses of some kind. Other sustained cells in the slice had offset rebound responses or neither rebound nor build-up. We had two cells with very noticeable rebound PSTH patterns (1079R and 466L) and others with neither rebound nor build-up (1079L, 339L, 351L, 325L, 939L). The rebound cells in the slices were “flat, disk-shaped” cells and in our material we described the cells with rebound as medium sized, one with disk-shaped dendrites and the other quite similar, but with elongated shape. Both cells were wider than the criteria used by us and others to label a cell as “flat.”

We have drawn attention to some correlations between small groups of cells with similar morphologies and similar physiological profiles; however, a major drawback in our study and others is that they are relatively under powered given the permutations of morphologies and responses. The use of brain slices from a knock-in mouse allows specific cell types to be targeted and the correlation of dendritic morphology with membrane properties (Ono et al., 2005), but even this approach is limited because it is not possible to trace the axonal output or the physiological inputs. Thus we are still in a position of having to painstakingly piece together an overall picture based on many studies each of which has a very limited set of data.

The IC is the site of termination of numerous projections from the brainstem (Brunso-Bechtold et al., 1981) and receives descending inputs from the cortex (Winer, 2005). It is considered to be a major site of reintegration of information processed at lower levels and some authors have even suggested that it is both the last level of analysis of the acoustical properties of sounds and also the level at which these are beginning to be formed into auditory objects (Nelken, 2008). The widespread ramification of the axons of the cells within the fibro-dendritic laminae detailed here and elsewhere provides a rich neuronal framework for such integration.

## Conflict of Interest Statement

The authors declare that the research was conducted in the absence of any commercial or financial relationships that could be construed as a potential conflict of interest.
